# Primate Simplexviruses Differ in Tropism for Macaque Cells

**DOI:** 10.3390/microorganisms11010026

**Published:** 2022-12-21

**Authors:** Heike Hofmann-Winkler, Abdul Rahman Siregar, Nesil Esiyok, Ignacio Rodríguez-Polo, Sabine Gärtner, Rüdiger Behr, Stefan Pöhlmann, Michael Winkler

**Affiliations:** 1 Infection Biology Unit, German Primate Center-Leibniz Institute for Primate Research, 37077 Göttingen, Germany; 2 Faculty of Biology and Psychology, University Göttingen, 37073 Göttingen, Germany; 3 Faculty of Biology, Universitas Gadjah Mada, Yogyakarta 55281, Indonesia; 4 Platform Degenerative Diseases, German Primate Center-Leibniz Institute for Primate Research, 37077 Göttingen, Germany; 5 DZHK (German Centre for Cardiovascular Research), Partner Site Göttingen, 37075 Göttingen, Germany

**Keywords:** herpes simplex virus type 1, Macacine alphaherpesvirus 1, Cercopithecine alphaherpesvirus 2, Papiine alphaherpesvirus 2, tropism, rhesus macaque, non-human primate, iPS cell, neural aggregate

## Abstract

Primate simplexviruses are closely related neurotropic herpesviruses, which are largely apathogenic in their respective host species. However, cross-species transmission of Macacine alphaherpesvirus 1 (McHV1, also termed herpes B virus) from rhesus macaques to humans can cause fatal encephalomyelitis. In contrast, closely related viruses, such as Cercopithecine alphaherpesvirus 2 (CeHV2, also termed simian agent 8) or Papiine alphaherpesvirus 2 (PaHV2, also termed herpesvirus papio 2), have not been linked to human disease and are believed to be largely apathogenic in humans. Here, we investigated whether McHV1, PaHV2 and CeHV2 differ in their capacity to infect human and non-human primate (NHP) cells. For comparison, we included the human simplexviruses HSV1 and HSV2 in our analyses. All five viruses replicated efficiently in cell lines of human and African green monkey origin, and McHV1 and PaHV2 also showed robust replication in rhesus macaque cell lines. In contrast, the replication of CeHV2 and particularly HSV1 and HSV2 in cell lines of rhesus macaque origin were reduced or inefficient. Similarly, McHV1, but not CeHV2, efficiently infected rhesus macaque brain organoids. These results point towards the previously unappreciated partial resistance of certain rhesus macaque cells to HSV1/HSV2/CeHV2 infection and reveal similarities between the cell tropism of McHV1 and PaHV2 that might be relevant for risk assessment.

## 1. Introduction

Simplexviruses of primates co-evolved with their respective hosts [1,2]. They share a common genome structure, which is essentially collinear with human herpes simplex virus type 1 (HSV1; Human alphaherpesvirus 1), the best-characterized species of this group. Several simplexviruses from non-human primates (NHP) have been isolated, including Macacine alphaherpesvirus 1 (McHV1, herpes B virus) [3], Cercopithecine alphaherpesvirus 2 (CeHV2, simian agent 8) [4], Papiine alphaherpesvirus 2 (PaHV2, herpesvirus papio 2) [5] and Panine alphaherpesvirus 3 (chimpanzee herpesvirus) [6]. Genome sequencing has revealed the conservation of all genes among these viruses, with the notable lack of the RL1 (γ34.5) gene in the genomes of McHV1, CeHV2 and PaHV2 [5,7,8,9,10].

The biology of the simplexvirus infection of NHP is believed to be similar to the infection of humans with HSV1, with a largely asymptomatic primary infection followed by lifelong viral latency in sensory neurons and occasional lesions due to reactivation [11]. This notion is mostly supported by studies analyzing McHV1 infection of macaques kept in captivity [12,13]. In addition to intraspecies transmission, cross-species transmission has been documented, especially when different NHP species were cohoused. In many cases, such transmission events (e.g., for McHV1) have been recognized because of apparent or fatal disease [14,15], but asymptomatic infections have also been documented [16]. Notably, the transmission of McHV1 from rhesus macaques to humans leads to encephalomyelitis with a high case-fatality rate [17]. In contrast, the transmission of CeHV2 and PaHV2 to humans has not been reported, despite these viruses being 79–86% identical to McHV1 on the genome level [10], and it is the general assumption that these viruses do not cause disease in humans.

Cell culture and animal studies highlight the potential of primate simplexviruses for cross-species transmission. Thus, HSV1 has been reported to replicate in cell lines from species as diverse as humans, NHP, hamsters and mice [18]. In addition, several mammalian species served as animal models for primate simplexviruses. Infection of mice is a common model to study the neuropathogenicity of primate simplexviruses [19,20,21,22], and rabbits and guinea pigs have been employed to study latency by HSV1 and McHV1 [23,24]. Recently, the use of rhesus macaques as an animal model for HSV1 [25,26,27,28] and HSV2 [29] infection has been reported. In contrast, older studies did not detect appreciable replication of HSV2 in macaques [30] and reported poor replication of HSV1 and HSV2 in macaque cell lines [31,32,33]. However, a systematic comparison of virus replication in cell lines of different primate origins is lacking. Therefore, we investigated the capacity of CeHV2, PaHV2 and McHV1 to infect cell lines of NHP and human origin. We report that McHV1 and PaHV2 replicated robustly in rhesus macaque cell lines, while the replication of HSV1, HSV2 and CeHV2 was inefficient.

## 2. Materials and Methods

### 2.1. Cell Culture

Cell lines 293T (DSMZ ACC 635) [34], A549 (ATCC CCL-185) [35], U251 (U373 MG) (ATCC HTB-17; kind gift by T. Stamminger) [36], LLC-MK2 (ATCC CCL-7) [37], sMAGI (NIH ARP5033) [38], TeloRF (kind gift by S. Voigt) [39,40], Vero76 (ATCC CRL-1587; kind gift by A. Maisner) [41] and Cos7 (ATCC CRL-1651) [42] were cultivated in DMEM supplemented with 10% FCS and Pen/Strep. Human cell lines were authenticated by STR typing following a published protocol [43]. The species identity of primate cell lines was authenticated by sequencing part of the mitochondrial CytB gene after PCR amplification [44].

Rhesus macaque induced pluripotent stem cell lines (iPSC lines) used as input cells for the neural aggregates were reported by Stauske et al. [45] and maintained as described. The pluripotent state and identity of the iPSC lines were regularly controlled.

### 2.2. Rhesus Macaque Neural Aggregate Generation and Culture

Neural aggregates were generated in stationary conditions following a protocol adapted from Lancaster et al. and Mansour et al. [46,47]. In brief, iPSCs were dissociated into single cells using Accutase, and 10,000 cells per well were transferred into 96-well ultra-low attachment plates in UPPS culture medium [45]. The medium was supplemented with 5 μM of pro-survival compound (ROCK2 inhibitor; Calbiochem DDD00033325) for the first 24 h. On day 3, embryoid bodies were transferred to Neural Induction Medium (NIM) (DMEM/F-12 (1:1), N2 Supplement, 20% KnockOut Serum, 3% Fetal Bovine Serum, 1% non-essential amino acids, and 2 mM GlutaMAX). The NIM was supplemented with 1 μg/mL heparin, 200 μM L-Ascorbic acid, 10 ng/mL of bFGF2, 10 μM SB431542, 2.5 μM dorsomorphine, and 1 mM sodium pyruvate for the first 4 days; and without heparin, bFGF, and sodium pyruvate for the subsequent 4 days with medium change every other day. On day 11, the neurospheres were embedded in Matrigel (10 mg/mL). After removing the culture medium, 50 μL of Matrigel drops were added on top of the neurospheres and allowed to polymerize for 20 min at 37 °C. After incubation, the Matrigel-embedded neurospheres were transferred to 48-well plates coated with anti-adherent rinsing solution in cerebral differentiation medium I (CDM I) (DMEM/F12: Neurobasal Medium (1:1), N2 supplement, B27 supplement without vitamin A, 1% non-essential amino acids, 2 mM GlutaMAX, and 2.8 ng/mL insulin). The CDM I was supplemented with 20 ng/mL bFGF and 20 ng/mL EGF and the medium was changed every other day. After 5 days, B27 supplement was added for the subsequent 7 days (CDM II) with medium change every other day. From day 22 onwards, EGF and FGF2 were replaced with 20 ng/mL BDNF and 20 ng/mL NT3 (CDM III). The medium was changed every other day. After generation, neural aggregates were used for characterization and infection experiments between days 70 and 100 of differentiation.

### 2.3. Viruses

HSV1 strain 17syn* and HSV2 strain 333 were a kind gift by Wali Hafezi (Institute of Virology, University Hospital Münster). CeHV2 (SA8) strain B264 and PaHV2 (HVP2) strain X313 were a kind gift by David Brown and Matthew Jones (Public Health England). McHV1 (herpes B virus) was a kind gift by Christiane Stahl-Hennig (German Primate Center). The viruses were propagated on Vero76 cells by infection at MOI 0.01 and harvested after the extensive cytopathic effect had developed.

### 2.4. Viral Replication Kinetics and Titration

For one-step growth curves, Vero76, A549, LLC-MK2 and TeloRF cells were seeded in 24-well plates at 60,000 cells/mL. On the next day, cells were infected with MOI 1 of the respective viruses. For this, the medium was replaced with 500 µL inoculum. After 1 h incubation at 37 °C, the inoculum was removed, cells were washed with PBS and finally incubated with 500 µL culture medium. At certain time points after infection, cell culture supernatant was harvested and centrifuged at 4000× *g* rpm for 5 min to pellet floating cells, and the cleared supernatant was frozen at −80 °C. To quantify cell-associated virus, infected cells were detached with Accutase, centrifuged at 4000× *g* rpm for 5 min, and the cell pellets were resuspended in 500 µL culture medium. The virus was released from cells with three freeze–thaw cycles followed by the removal of cellular debris by centrifugation at 4000 rpm for 5 min. The resulting supernatant was used for titrations.

Virus titrations were uniformly carried out on Vero76 cells, which were seeded in 24-well plates at 100,000 cells/well. On the next day, the culture medium was removed, and cells were infected with virus supernatant in 10-fold dilutions for 1 h at 37 °C. Thereafter, inoculum was removed and replaced with Avicel overlay medium (2 vol. culture medium mixed with 1 vol. 3% Avicel; FMC, Philadelphia, PA, USA) [48]. After incubation for 2–4 days, depending on the virus, the medium containing Avicel was removed, cells were washed two times with PBS and then fixated by using cold methanol for 15 min at −20 °C. For the visualization of plaques, cells were stained with a crystal violet solution (1 g crystal violet, 100 mL ethanol in a final volume of 500 mL water), followed by one wash with water.

### 2.5. Infection of Neural Aggregates

The infection of neural aggregates was performed in 24-well plates in a volume of 500 µL CDMIII medium containing the virus. Based on a mean surface area of 11–13 mm^2^ (diameter 1.9–2.1 mm), we estimated that there would be approximately 2–3000 cells on the surface of the neural aggregates. Therefore, we chose to infect with 2000 pfu of either McHV1 or CeHV2, reflecting an MOI 0.5–1. The inoculum was diluted in CDMIII medium. For mock control, neural aggregates were incubated with fresh CDMIII medium without the virus. After an incubation of 1 h in a cell culture incubator (37 °C, 80% humidity, 5% CO_2_), the inoculum was removed, and neural aggregates were washed in 500 µL DMEM, followed by the addition of 500 µL CDMIII medium. At defined time points post-infection (1, 24, 48 and 72 h), culture supernatant was removed and replaced with fresh CDMIII medium and stored for subsequent virus titration. After titration, the cumulative titer was calculated for each neural aggregate for each time point.

### 2.6. Microscopy

For McHV1 infected samples, brightfield images were taken at 10× magnification on an Olympus IX70 using Cell^F software. For all other viruses, brightfield images were taken at 10× magnification using the ESID detector of a LSM800 (Zeiss, Oberkochen, Germany) microscope and ZEN software (version 2.3). Images were adjusted in ImageJ [49] to cover the same area.

### 2.7. Immunohistochemistry

Neural aggregates (70–100 days old) were fixed in a 4% paraformaldehyde (PFA) solution for 20 min and washed 3 times with DPBS. Each fixed neural aggregate was then embedded in 2% agarose liquefied at 50 °C in 2 mL reaction tubes. Then agarose was chilled on ice for 5 to 10 min to allow the agarose to solidify. Embedded neural aggregates were transferred to 4% PFA in 2 mL reaction tubes for a second fixation and incubated overnight on a shaker. After three washes in DPBS, the neural aggregates were embedded in paraffin and sectioned at 3 µm.

For immunohistochemistry, neural aggregates were deparaffinized and rehydrated using xylol and progressively decreasing concentrations of ethanol. Antigen retrieval was performed by microwaving the sections in 10 mM sodium citrate buffer (pH 7.6) for 10 min. Endogenous peroxidase activity was inhibited by the peroxidase-blocking reagent. Anti-HSV1 + 2 polyclonal rabbit antibody (1:800) (DS-PB-00984, RayBiotech, Peachtree Corners, GA, USA), which recognized both CeHV2 and McHV1 in infected cell cultures [50], was used for the detection of viral proteins in McHV1-, CeHV2-, and mock-infected neural aggregates. Anti-βIII-tubulin monoclonal mouse antibody (1:50) (T8660; Sigma Aldrich, St. Louis, MO, USA) was used as a neuron marker. Anti-Rabbit IgG isotype was used for control stainings. The detection of the primary antibodies was carried out using Envision FLEX/HRP secondary antibody (GV80011-2; DAKO, Hamburg, Germany). 3,3′-diaminobenzidine (DAB) chromogen was used as the substrate for the HRP, and Mayer’s hemalum solution was used as the counterstain. Images of sections were taken using Aperio CS2 Slide Scanner and analyzed using Aperio ImageScope (Leica, Wetzlar, Germany) software.

For immunofluorescence staining, deparaffinization and antigen retrieval steps were performed as described above. The neural aggregate sections were blocked in 1% BSA in DPBS for 20 min at room temperature. After washing three times in DPBS, the sections were incubated (1 h at room temperature or overnight at 4 °C) with Anti-HSV1 + 2 (1:200) (NB120-9533; Novus Biologicals, Wiesbaden, Germany), which recognized McHV1 in infected cell cultures [50], and anti-βIII-tubulin (1:50) antibodies. Subsequently and after washing with PBS, secondary antibody incubation (1 h) was performed using AlexaFluor488™ goat anti-mouse IgG (Invitrogen, 1829920) (1:1000) and AlexaFluor555™ donkey anti-rabbit IgG (Invitrogen, 2180682) (1:1000). Incubation with DAPI (10 min, room temperature) (0.1 µg/mL) was used for nuclear stain. Stained sections were imaged using Zeiss Observer Z1 (Zeiss, Oberkochen, Germany) inverted fluorescence microscope and analyzed using ImageJ software (version 1.53t) [49].

## 3. Results

For a systematic analysis of the replication of human and NHP simplexviruses, we used two well-characterized human viruses, HSV1 and HSV2, as well as the primate simplexviruses McHV1, PaHV2 and CeHV2. Replication of these viruses was studied in cell lines generated from their respective host species, rhesus macaque (McHV1), African green monkey (CeHV2) and human (HSV-1 and HSV2), and all cell lines chosen had previously been used in infection experiments with different primate simplexviruses.

For the first experiment, we performed one-step growth curves to gain information on the replication kinetics in four cell lines. Vero76 epithelial cells, which were derived from the kidney of an African green monkey, were used as a positive control since all viruses tested are routinely propagated in these cells [5,9,10,25,28,29,51]. In addition, we used the human A549 epithelial lung adenocarcinoma cell line, which has been used for virus isolation and functional studies of HSV1 and HSV2 [52,53,54,55] and was reported to be permissive to CeHV2 infection [56]. Finally, we employed two cell lines from rhesus macaques, LLC-MK2 (epithelial kidney) [37] and TeloRF (TERT-immortalized skin fibroblast) [39]. LLC-MK2 cells were previously reported to support the replication of CeHV2 [57], while a CeHV2 reporter virus generated by us hardly grew in this cell line and also failed to grow efficiently in TeloRF cells [56]. To monitor virus replication, supernatants and cells from infected cultures were harvested over the course of 72 h, and virus titers were determined by plaque assay.

**Figure 1:**
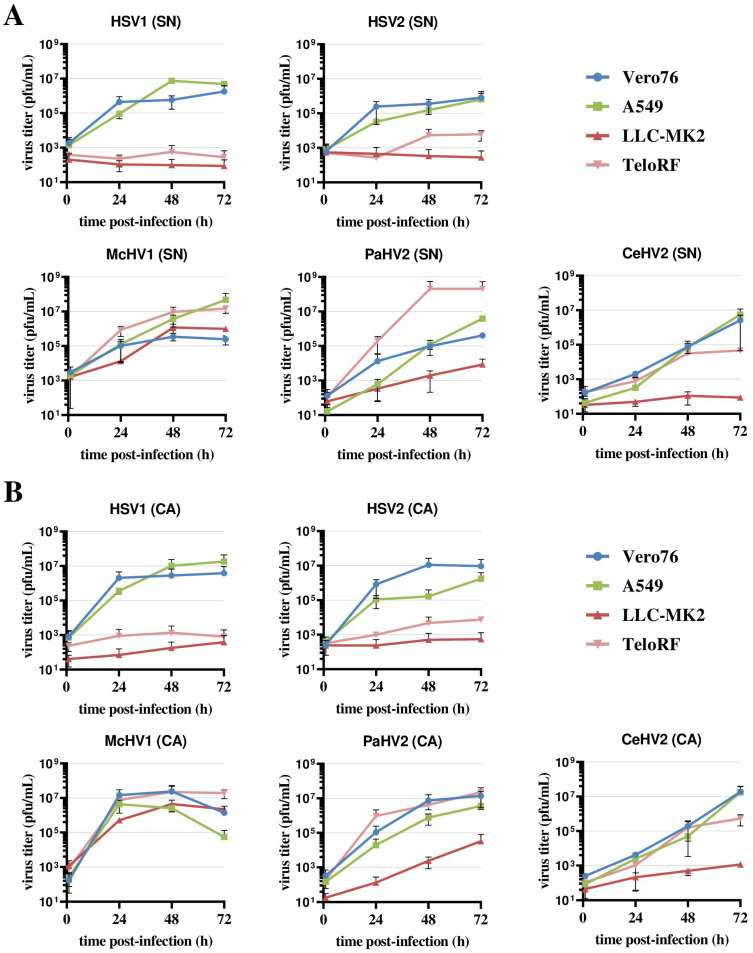
**Comparison of primate simplexvirus replication in primate cells lines.** Cell lines of human (A549), rhesus macaque (LLC-MK2, TeloRF) and African green monkey (Vero76) origin were infected with five primate simplexviruses at MOI1: Herpes simplex viruses type 1 (HSV1) and type 2 (HSV2), Macacine alphaherpesvirus 1 (McHV1), Papiine alphaherpesvirus 2 (PaHV2) and Cercopithecine alphaherpesvirus 2 (CeHV2). Virus titers from supernatant (SN, panel (**A**)) or infected cells (CA, cell associated; panel (**B**)) harvested at the indicated time points were determined by plaque assay on Vero76 cells. The average of two independent experiments carried out with triplicate samples are shown. Error bars indicate standard error of the mean, SEM.

Vero76 and A549 cells supported the efficient replication of all five simplexviruses, regardless of whether the supernatant or cell-associated virus was analyzed (Figure 1A,B). The highest titers were measured for McHV1 and HSV1, while CeHV2 titers were still increasing at 72 h post-infection (hpi). The two rhesus macaque cell lines, LLC-MK2 and TeloRF, supported efficient replication of McHV1 and PaHV2, although the replication of PaHV2 on LLC-MK2 cells was reduced relative to the other cell lines tested. In contrast, the replication of HSV1 and HSV2 was inefficient on both cell lines (Figure 1). CeHV2 replicated poorly on LLC-MK2 cells, while replication on TeloRF cells was robust, but titers at 72 h post-infection (hpi) were reduced as compared to PaHV2 and McHV1. Finally, titers determined from virus-containing culture supernatants and cell-associated viruses were comparable. Thus, the simplexviruses studied could be grouped into viruses that replicated well on both rhesus macaque cell lines tested (McHV1), viruses that failed to replicate efficiently in these cell lines (HSV1, HSV2), and viruses with an intermediate phenotype (PaHV2 and CeHV2), with PaHV2 showing a somewhat increased replicative capacity in rhesus macaque cell lines relative to CeHV2.

**Figure 2:**
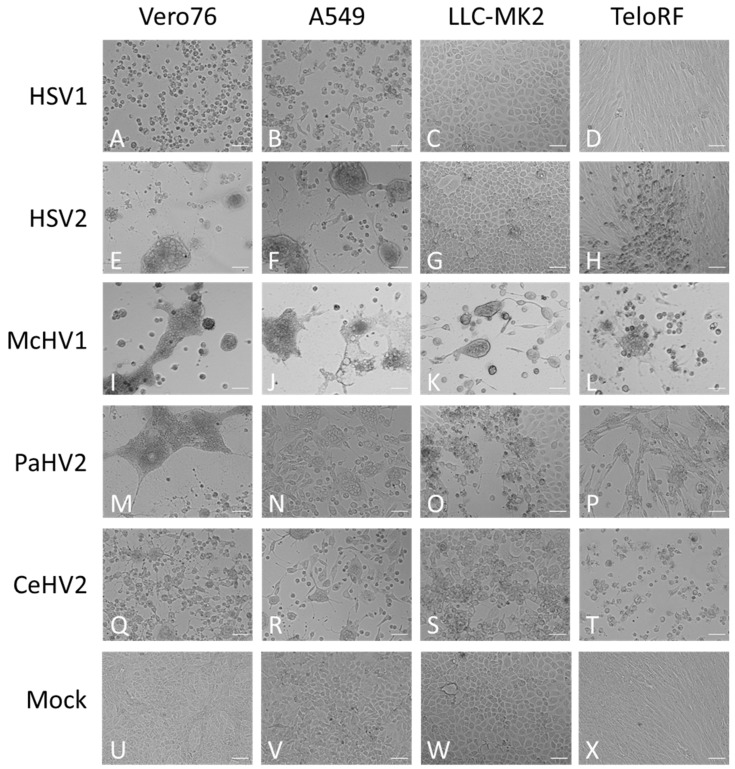
**Morphology of infected cells.** African green monkey Vero76 (**A**,**E**,**I**,**M**,**Q**,**U**), human A549 (**B**,**F**,**J**,**N**,**R**,**V**) and rhesus macaque LLC-MK2 (**C**,**G**,**K**,**O**,**S**,**W**) and TeloRF (**D**,**H**,**L**,**P**,**T**,**X**) cell lines were infected with five primate simplexviruses at MOI1: Herpes simplex viruses type 1 (HSV1) (**A**–**D**) and type 2 (HSV2) (**E**–**H**), Macacine alphaherpesvirus 1 (McHV1) (**I–L**), Papiine alphaherpesvirus 2 (PaHV2) (**M**–**P**) and Cercopithecine alphaherpesvirus 2 (CeHV2) (**Q**–**T**) Brightfield images of infected or mock (**U**–**X**) cultures were taken at 72 hpi at 10× magnification. The scale bar indicates 100 µm.

Next, we investigated whether replication efficiency correlated with the formation of cytopathic effects (CPE). The typical morphologies of the cell lines are shown in Figure 2U–X. Cell rounding, detachment and syncytia formation were detected as early as 24 hpi and increased up to 72 hpi (Figure 2 and data not shown) and were dependent on the virus and cell line. McHV1 induced the formation of large syncytia in all cell lines tested (Figure 2I–L), in keeping with robust replication (Figure 1). HSV2 also induced the formation of large syncytia in Vero76 and A549 cells (Figure 2E,F) but not in rhesus macaque cell lines (Figure 2G,H), again in keeping with its replicative potential in these cell lines. Similar findings were made for HSV1, although mainly cell rounding and detachment rather than syncytia formation was observed (Figure 2A–D). Finally, CeHV2 and PaHV2 caused detachment and cell rounding to a similar extent in all cell lines tested, with the exception of Vero76 cells, in which PaHV2 but not CeHV2 induced large syncytia (Figure 2M–T). In sum, CPE induction largely matched the replicative capacity of the primate simplexviruses tested.

**Figure 3:**
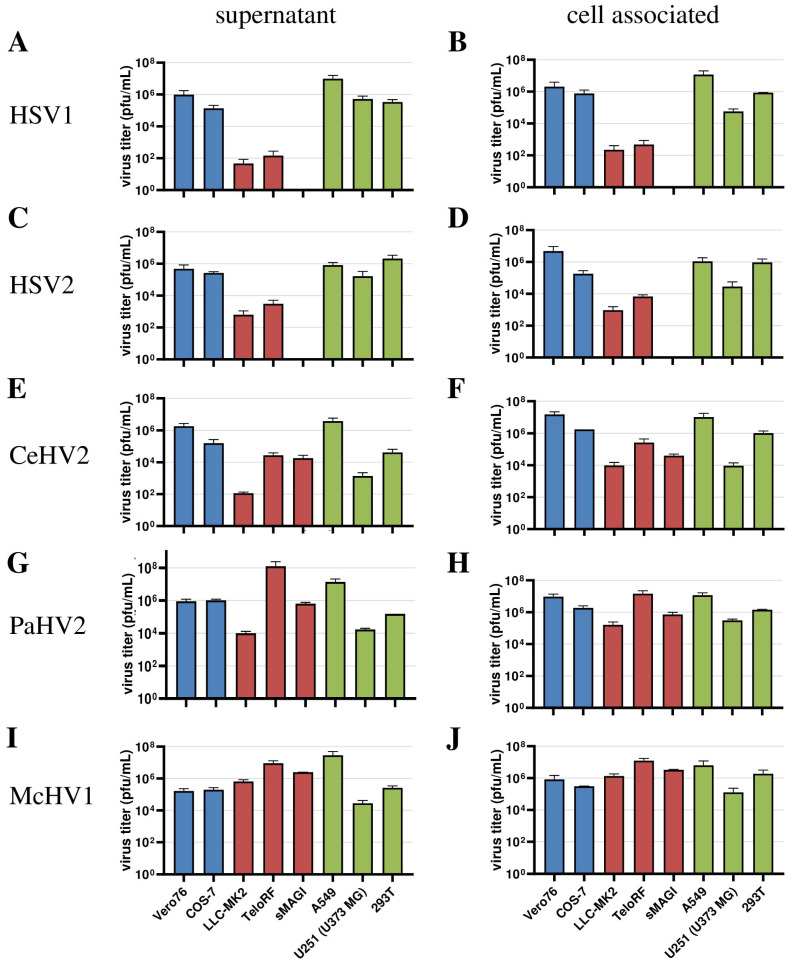
**Comparison of virus production in a panel of primate cell lines.** Herpesviruses HSV1 (**A**,**B**), HSV2 (**C**,**D**), CeHV2 (**E**,**F**), PaHV2 (**G**,**H**) and McHV1 (**I**,**J**) were used to infect human (A549 epithelial lung adenocarcinoma, U251 (U373 MG) glioblastoma, 293T epithelial kidney), rhesus macaque (LLC-MK2 epithelial kidney, TeloRF skin fibroblast, sMAGI epithelial mammary gland) and African green monkey (Vero76 epithelial kidney, Cos7 fibroblast kidney) cell lines with MOI1. Virus titers from supernatants (**A**,**C**,**E**,**G**,**I**) and infected cells (**B**,**D**,**F**,**H**,**J**) were determined by plaque assay on Vero76 cells. The results of two to four independent experiments carried out with triplicate samples are shown. Error bars indicate standard error of the mean, SEM.

Next, we extended our analysis to a larger panel of cell lines in order to determine whether our initial observations could be corroborated. For this, we included the human cell lines 293T (epithelial kidney) and U251 (U373 MG, glioblastoma) in our analyses, which are both established in simplexvirus research [58,59]. In addition, we analyzed African green monkey-derived Cos-7 kidney fibroblast-like cells and rhesus macaque-derived sMAGI cells (epithelial mammary gland) as additional NHP cell lines. In this experiment, analysis was performed at 72 h post-infection since our initial experiment (Figure 1) revealed that the titers of most viruses reached their plateau at this time point regardless of the cell line used. We found that HSV1 and HSV2 were unable to replicate in sMAGI cells while replication in all cell lines of human and African green monkey origin was efficient (Figure 3A–D), consistent with diverse rhesus macaque cell lines being partially resistant against HSV1 and HSV2 infection. In contrast, McHV1 infected all cell lines with high efficiency (Figure 3I,J). Further, CeHV2 and PaHV2 continued to show an intermediate phenotype regarding the infection of rhesus macaque cell lines, which represent different cell types and originated from different tissues, with sMAGI cell infection by PaHV2 being more efficient than infection by CeHV2 (Figure 3E–H). Finally, no major differences were observed when analyzing cell-free and cell-associated viruses. These results confirmed that rhesus macaque cell lines might be partially resistant against HSV1, HSV2 and likely CeHV2 infection.

**Figure 4:**
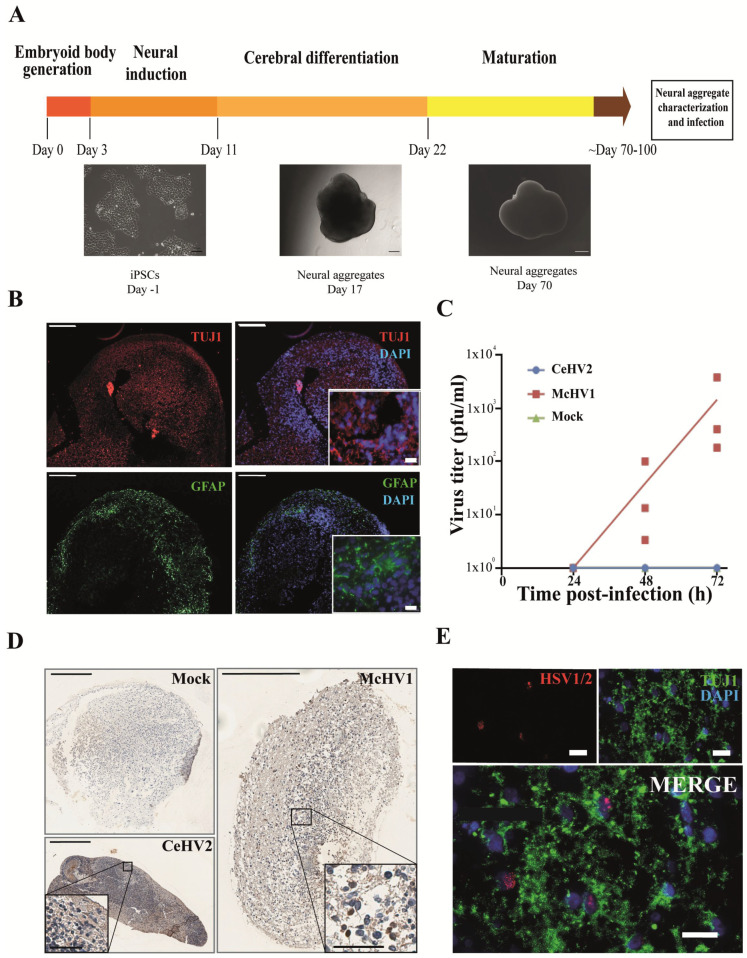
**Comparison of McHV1 and CeHV2 infection of rhesus macaque neural cell aggregates.** (**A**) Schematic representation of the neural aggregate generation protocol. Neural aggregates were derived from rhesus macaque iPSCs and cultured for up to 100 days. Representative images of neural aggregates on day 17 and day 70 are shown. Scale bars: 100 µm, 200 µm, and 500 µm, respectively. (**B**) Neural aggregates were positive for a neuronal (TUJ1) and a glial marker (GFAP) at day 70. Scale bars: 200 µm; inset, 20 µm. (**C**) Rhesus macaque neural aggregates were infected in triplicates with 2000 pfu of McHV1 or CeHV2, respectively, or mock treated. Virus titers from supernatants of neurospheres were titrated on Vero76 cells. Dots show titers for individual organoids, while lines show the mean of three organoids. The results were confirmed in an independent experiment. (**D**) Immunohistochemical staining of the mock-treated and CeHV2- or McHV1-infected neural aggregates using an HSV1/2 reactive rabbit polyclonal serum (RayBiotech) that detects CeHV2 and McHV1 in infected cell cultures. Infected cells were found only in the McHV1-infected neural aggregates (inset images). Scale bars: 400 µm; inset, 200 µm. (**E**) Fluorescence imaging of McHV1-infected neural aggregates for TUJ1 (neuronal marker) and viral antigen, using rabbit polyclonal (Novus), which recognizes McHV1 in infected cell cultures, depicting viral proteins localized in nuclei of TUJ1+ cells. Scale bars: 20 µm.

Finally, we investigated whether the suspected reduced permissiveness of rhesus macaque cells for CeHV2 as compared to McHV1 infection could be confirmed in a more relevant cell system. For this, we infected neuronal cells in a rhesus macaque 3D neural aggregate model. The model was based on rhesus macaque induced pluripotent stem cells (iPSCs). The 3D differentiation protocol was established according to published reports for human brain organoid generation [46,47] (Figure 4A). Successful neural induction was assessed in the neural aggregates after 70–100 days of differentiation by staining with general markers for neurons and glia cells (Figure 4B). The aggregates contained neuron- and glia-like cells, assessed by immunostaining for cell-specific markers TUJ1 (for neurons) and GFAP (for glial cells), respectively (Figure 4B).

Notably, McHV1 productively infected the neurospheres, while CeHV2 did not (Figure 4C). Immunohistochemical staining indicated that numerous individual cells were infected with McHV1 throughout the neurosphere at 72hpi, as evidenced by intense staining of compact cells (Figure 4D). In CeHV2-infected neurospheres, we observed some background staining but no staining as seen for McHV1, in agreement with infection experiments (Figure 4C) showing that CeHV2 was unable to infect neurospheres. Fluorescence imaging of the McHV1-infected neural aggregates confirmed the presence of McHV1 proteins in the nuclei of TUJ1+ neurons within the neural aggregates (Figure 4E). Thus, McHV1, but not CeHV2, seems to have a high capacity to infect rhesus macaque neural cells.

## 4. Discussion

Simplexviruses exhibit a broad species tropism, being able to infect many mammalian species, from mice to humans [18]. Thus, when we established reporter viruses for CeHV2 and tested replication in cell lines from different species, it came as a surprise that only very limited virus production was detected in several cell lines derived from rhesus macaques [56]. Although an older report using wildtype CeHV2 came to conflicting conclusions [57], several publications have also reported limited or no replication of HSV1 and HSV2 in rhesus macaque cells [31,32,33]. However, a comparative analysis has been lacking so far. Our comparison of five human and NHP simplexviruses in cell lines of human and NHP (rhesus macaque and African green monkey) origin shows that HSV1, HSV2, and, to some degree, CeHV2 have a limited capacity to infect rhesus macaque cell lines. Importantly, the cell lines tested represent different cell types (epithelial, fibroblast) and tissues (kidney, mammary gland, skin), making it likely that differential infection reflects differential species tropism. In contrast, PaHV2 and particularly McHV1 infected these cell lines efficiently, a finding that confirms and extends previous studies [32,60,61]. Importantly, studies with a rhesus macaque neural cell aggregate model demonstrated that the reduced capacity of CeHV2 to infect rhesus macaque cell lines extended to neural cells grown in a 3D culture system. In contrast, McHV1 replicated efficiently in this cell system, in agreement with observations for HSV1 in human brain organoids [62,63]. Collectively, we observed a differential capacity of primate simplexviruses to infect rhesus macaque cells.

We note a minor difference between our present and previous findings. Using wildtype CeHV2, we did not observe the strongly reduced replication in rhesus macaque cell lines that we had previously recorded for a CeHV2 reporter virus [56]. These differences can be related to the fusion of ICP4 with a reporter gene [50], which leads to reduced virus production, most likely due to impaired ICP4 expression. Regardless of the reasons for this discrepancy, it should be noted that replication of CeHV2 was still reduced by roughly 2–4 log compared to McHV1 and PaHV2 in rhesus macaque cell lines, underlining the differences in the capacity of NHP herpesviruses to replicate in rhesus macaque cells.

All viruses showed CPE in permissive cell lines, while the extent of syncytia formation differed between the individual viruses. For human simplexviruses, the extent of syncytia formation was strain dependent and mostly reflects a cell culture adaption, which may also be cell line-dependent [64]. Thus, syncytia-forming viruses are rapidly selected for in cell culture, while this phenotype does not impact virus titers [65]. Thus, propagation in cell culture can lead to adaption due to the selection of preexisting variants. Earlier studies suggested that HSV1 and HSV2 may become adapted to rhesus monkey or baby hamster kidney cells upon continued propagation [33]. However, the underlying molecular reason for these adaptions is not yet known [66]. All viruses in our study have been extensively passaged on Vero cells [5,9,10,51] and likely have adapted to these cells. However, despite this common adaption, these viruses show clear differences in their ability to infect cells derived from rhesus macaques. We are therefore convinced that differences in the tropism for macaque cells cannot be explained by adaption to Vero cells.

The nature of the block to efficient infection of rhesus macaque cell lines with HSV1, HSV2 and CeHV2 remains to be elucidated. The entry of HSV1 into target cells is well studied [67,68] and encompasses the interaction of two glycoproteins, gD and gB, with multiple cellular receptors. Presently, little is known about the receptor usage of primate simplexviruses, although it has been shown that McHV1 can use human Nectin-1 but not the herpesvirus entry mediator or immunoglobulin-like type 2 receptor alpha for entry [69,70]. In the absence of knowledge on species-specific glycoprotein-receptor interactions, it is difficult to judge whether glycoprotein-receptor interactions are responsible for the differential susceptibility of rhesus macaque cell lines to infection with primate simplexviruses. Apart from glycoprotein receptor interactions, restriction factors of the innate immune system might also modulate permissiveness to infection. In fact, TRIM5α of rhesus macaque origin has been reported to reduce infection by HSV1 and HSV2 [71]. However, similar effects were also reported for African green monkey TRIM5α, making TRIM5α an unlikely candidate to explain the relative resistance of rhesus macaque cells to CeHV2 and particularly HSV1 and HSV2 infection, and a yet unidentified restriction factor might be responsible.

McHV1 can cause severe disease in humans and requires handling in BSL3 laboratories in Germany and BSL4 laboratories in the US. In contrast, CeHV2 and PaHV2 are believed to constitute a moderate threat to humans. The present study does not provide evidence that this concept should be changed. However, our finding that PaHV2 more closely resembles McHV1 than CeHV2 regarding the infection of rhesus macaque cells might hint towards biological similarities between McHV1 and PaHV2. Indeed, for both McHV1 and PaHV2, neurovirulence in mice has been demonstrated, while CeHV2 was avirulent [21,22,72], suggesting that risk assessment for PaHV2 at some point might need to be revisited.

## Data Availability

The data presented in this study are available on request from the corresponding author.
